# The proton pump inhibitor pantoprazole disrupts protein degradation systems and sensitizes cancer cells to death under various stresses

**DOI:** 10.1038/s41419-018-0642-6

**Published:** 2018-05-22

**Authors:** Yu Cao, Min Chen, Dehua Tang, Hongli Yan, Xiwei Ding, Fan Zhou, Mingming Zhang, Guifang Xu, Weijie Zhang, Shu Zhang, Yuzheng Zhuge, Lei Wang, Xiaoping Zou

**Affiliations:** 1 Department of Gastroenterology, Drum Tower Hospital Affiliated to Medical School of Nanjing University, Nanjing, Jiangsu Province China; 2 Jiangsu Clinical Medical Center of Digestive Disease, Nanjing, China; 3 Department of Laboratory Medicine, Changhai Hospital, the Second Military Medical University, Shanghai, China; 4 Department of General Surgery, Drum Tower Hospital, Medical School of Nanjing University, Nanjing, Jiangsu Province China; 5 Department of General Surgery, Drum Tower Clinical College of Nanjing Medical University, Nanjing, Jiangsu Province China

## Abstract

Proton pump inhibitors (PPIs) play a role in antitumor activity, with studies showing specialized impacts of PPIs on cancer cell apoptosis, metastasis, and autophagy. In this study, we demonstrated that pantoprazole (PPI) increased autophagosomes formation and affected autophagic flux depending on the pH conditions. PPI specifically elevated SQSTM1 protein levels by increasing SQSTM1 transcription via NFE2L2 activation independent of the specific effect of PPI on autophagic flux. Via decreasing proteasome subunits expression, PPI significantly impaired the function of the proteasome, accompanied by the accumulation of undegraded poly-ubiquitinated proteins. Notably, PPI-induced autophagy functioned as a downstream response of proteasome inhibition by PPI, while suppressing protein synthesis abrogated autophagy. Blocking autophagic flux in neutral pH condition or further impairing proteasome function with proteasome inhibitors, significantly aggravated PPI cytotoxicity by worsening protein degradation ability. Interestingly, under conditions of mitochondrial stress, PPI showed significant synergism when combined with Bcl-2 inhibitors. Taken together, these findings provide a new understanding of the impact of PPIs on cancer cells’ biological processes and highlight the potential to develop more efficient and effective combination therapies.

## Introduction

Proteostasis is a necessity for cell survival when facing stress^1^. Two major protein degradation systems have developed to handle these tasks, the ubiquitin-proteasome system (UPS) and the autophagy-lysosome pathway (ALP)^2^. Proteasome inhibition caused poly-ubiquitinated proteins accumulation, and then activated autophagy to eliminate protein aggregates^1–6^. UPS and ALP share common signaling receptors and substrates such as SQSTM1^7^. Therefore, in the context of proteasome inhibition, the complexity of using SQSTM1 as an autophagy marker should be underscored^8,9^. Besides autophagy, accumulation of unfolded proteins in the endoplasmic reticulum (ER) upon proteasome inhibition, initiates a specialized response known as the unfolded protein response (UPR)^10^. The intensity of UPR reflects the protein overload stress. Once beyond the scope of tolerance, a terminal UPR was provoked and the irreversible damage would be brought to cancer cells under integrated stress^11^.

Mitochondrial permeabilization is controlled by the balance of antiapoptotic and proapoptotic Bcl-2 family proteins, which set the apoptotic threshold^12^. In the case of proteasome inhibition, there would be a complex crosstalk between mitochondria and other organelles, and various regulations of Bcl-2 family proteins^13,14^. Silencing the prosurvival pathways by Bcl-2 inhibitors would make cancer cells under integrated stress more sensitive to death^14^. Proteasome inhibitors have been confirmed exerting a synergistic cytotoxicity when combined with Bcl-2 inhibitors^15–17^.

Previous works have reported the inhibitory effects of proton pump inhibitors (PPIs) on autophagy in low pH condition, which makes PPI transformed into the active molecule to inhibit the vacuolar-type H^+^-translocating ATPase (V-ATPase)^18–22^. Moreover, Marino et al.^19^ reported that in addition to blocking the autophagic flux in low pH condition, ESOM also induced the early accumulation of autophagosomes.Thus we are wondering whether PPI has similar impacts on autophagy in neutral pH condition. Besides autophagy, the impact of PPI on another protein degradation system remains to be investigated because there was crosstalk between the ubiquitin-proteasome and autophagy-lysosome systems. A dose-dependent and time-dependent apoptotic-like cytotoxicity by PPI has been confirmed in B-cell lymphoma^18^, melanoma^23^, and multiple myeloma^24^. The effect of PPI was associated with alkalinization of lysosomal pH and lysosomal membrane permeabilization. Whether PPI-induced cell death was caspase dependent or not depended on tumor histology^18,23,24^, suggesting that the specificity of the death pathway depended on the original cell type. Moreover, the impacts of PPI on Bcl-2 family members have not been investigated, and whether they were involved in PPI-induced apoptosis remains to be seen. We focused on gastric cancer cell lines for the study because our previous works^25,26^ about pantoprazole were about gastric cancer. In this study, at least five unexplored mechanisms have been discovered and studied. First, PPI consistently promoted autophagosome formation in both low pH and neutral pH conditions, with TM9SF4-mTOR pathway playing an important role. Second, PPI-induced autophagy with increased SQSTM1 transcription, which was mediated by oxidative stress induced-Nrf2 in both low pH and neutral pH conditions. Third, pantoprazole inhibits proteasome function via transcriptionally reducing proteasome subunits partially via inhibiting STAT3 independent of pH conditions, which contributes to the activation of UPR and ER stress. Fourth, proteasome inhibition or ER stress was responsible for the activation of PPI-induced autophagy. Last but not least, Bcl-2/Bcl-xl inhibitors such as ABT-263 and ABT-737 have synergistic interaction with PPI in gastric cancer cells in both pH 7.4 and pH 6.5 conditions, which has a broad prospect in the field of cancer treatment.

## Results

### PPI-induced autophagosome formation via TM9SF4-mTOR pathway

**Fig. 1:**
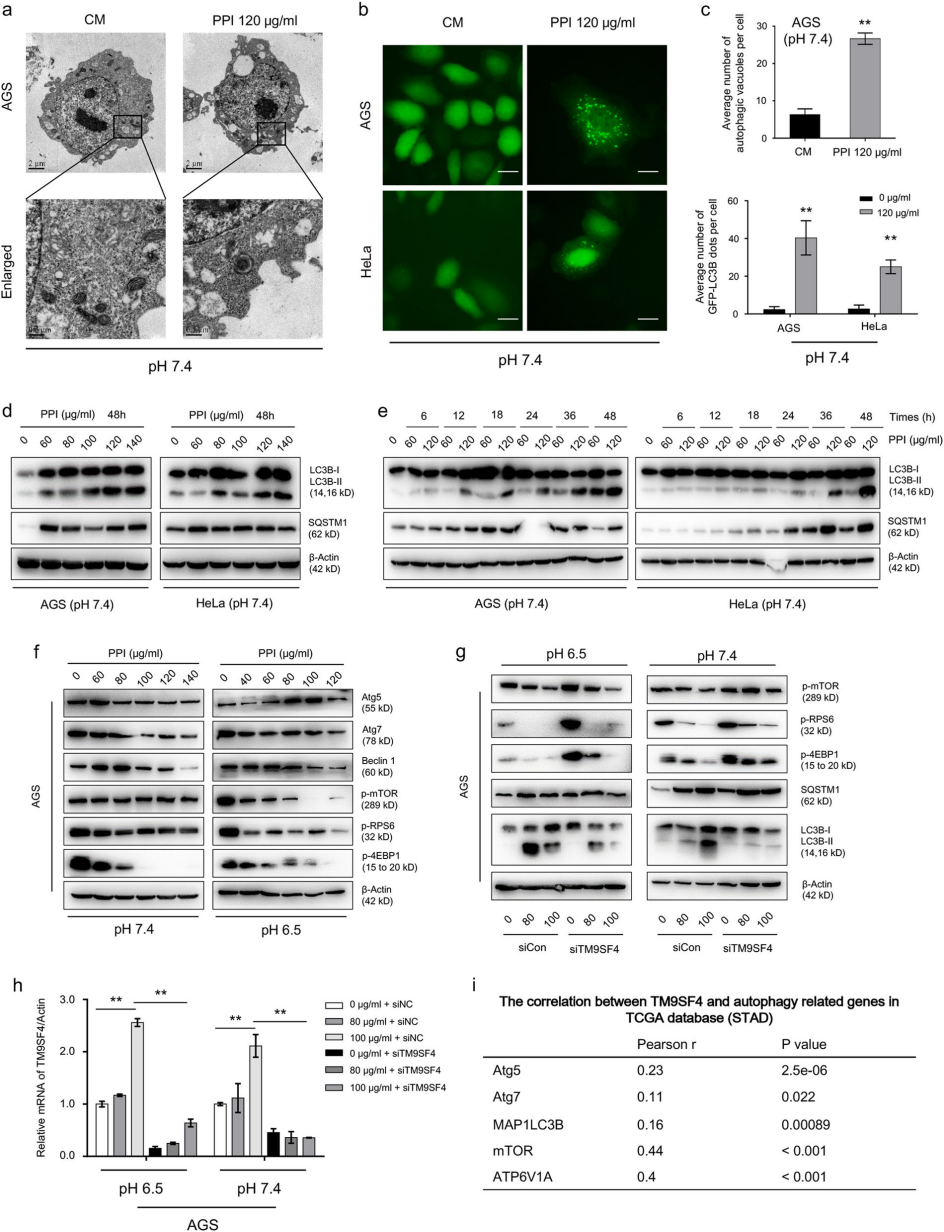
Pantoprazole induced autophagosome biogenesis via TM9SF4-mTOR pathway. **a** AGS cells treated with 120 μg/ml PPI for 48 h in pH 7.4 condition, were imaged by transmission electron microscopy. Representative micrographs showed increased abundance of lysosomes in cells after PPI treatment compared with control cells. Scale bar: 500 nm. **b** AGS and HeLa cells transfected with GFP-LC3B plasmid were treated with 120 μg/ml PPI for 48 h in pH 7.4 condition. Scale bar: 20 μm. **c** The number of autophagic vacuoles and GFP-LC3B dots in each cell were quantified. Data were presented as mean ± SD from three independent experiments (**p < 0.01, identified by Student’s *t*-test). **d**, **e** AGS and HeLa cells were treated for 48 h in pH 7.4 condition with indicated concentrations of PPI, or treated with 60 and 120 μg/ml PPI for the indicated times. Levels of LC3B-II and SQSTM1 protein were analyzed by western blot. β-actin served as an internal control. **f** mTOR pathway proteins levels in AGS cells after treated with PPI for 48 h both in pH 7.4 and 6.5 conditions, were detected by western blot analysis. **g**, **h** AGS cells were transfected with TM9SF4 siRNA for 48 h, and then incubated with 80 and 100 μg/ml PPI for another 48 h. The level of indicated proteins were analyzed by western blot (**g**). TM9SF4 knockdown efficiency was verified by Q-PCR (**h**). **i** Data showing the positive correlation between TM9SF4 and related autophagy genes mRNA expression in TCGA STAD corhort, were generated by GEPIA (http://gepia.cancer-pku.cn/detail.php?clicktag=correlation). Pearson’s coefficient tests were performed to assess statistical significance

The increased cytoplasmic vacuolation after PPI treatment was confirmed by transmission electron microscopy and GFP-LC3B puncta assay (Fig. 1a–c and Supplementary Figure S3a-c) in both low and neutral pH conditions. The transition from LC3B-I to LC3B-II was also enhanced in a dose-dependent manner and time-dependent manner (Fig. 1d–e and Supplementary Figure S3d), and real for different cancer cell lines (Supplementary Figure S1e). The key negative regulator of autophagy, mTOR signaling pathway and a number of autophagy regulators were significantly upregulated after PPI treatment (Fig. 1f, Supplementary Figure S2a and S2b, Supplementary Figure S3e and S3f)^27^. To determine whether they were responsible for PPI-induced autophagy, we adopted genetic and pharmacological inhibition approaches. After Atg5 and Atg7 were knocked down by siRNA, the PPI-induced LC3B-II accumulation was abolished (Supplementary Figure S2c-f and Supplementary Figure S3g). On the contrary, disrupting class III PI3K complex, neither by knocking down Beclin 1 via siRNA or inhibiting PI3K using wortmannin (WM), there was no difference (Supplementary Figure S2c and S2g, Supplementary Figure S3g). Autophagy was an important adaptation mechanism for cancer cells when exposed to an acidic environment^28^. Cancer cells under microenvironmental stress (including low pH) use to cannibalize other cells^29^. TM9SF4 has been recently reported as an important regulator of autophagy^30,31^ and cannibalism^29^. Suppression of TM9SF4 by siRNAs attenuated the PPI-stimulated dephosphorylation of mTOR, RPS6, and 4E-BP1 (Fig. 1g, h). More important, knocking down TM9SF4 reduced LC3B accumulation after PPI treatment via reversed mTORC1 activity. In TCGA cohort, the mRNA expression level of TM9SF4 was also significantly correlated with important autophagy genes (Fig. 1i). Taken together, these data showed that PPI induced autophagosome formation via TM9SF4-mTOR pathway, consistent with recent studies^30,32^.

### PPI didn’t always block the autophagic flux in neutral pH

**Fig. 2:**
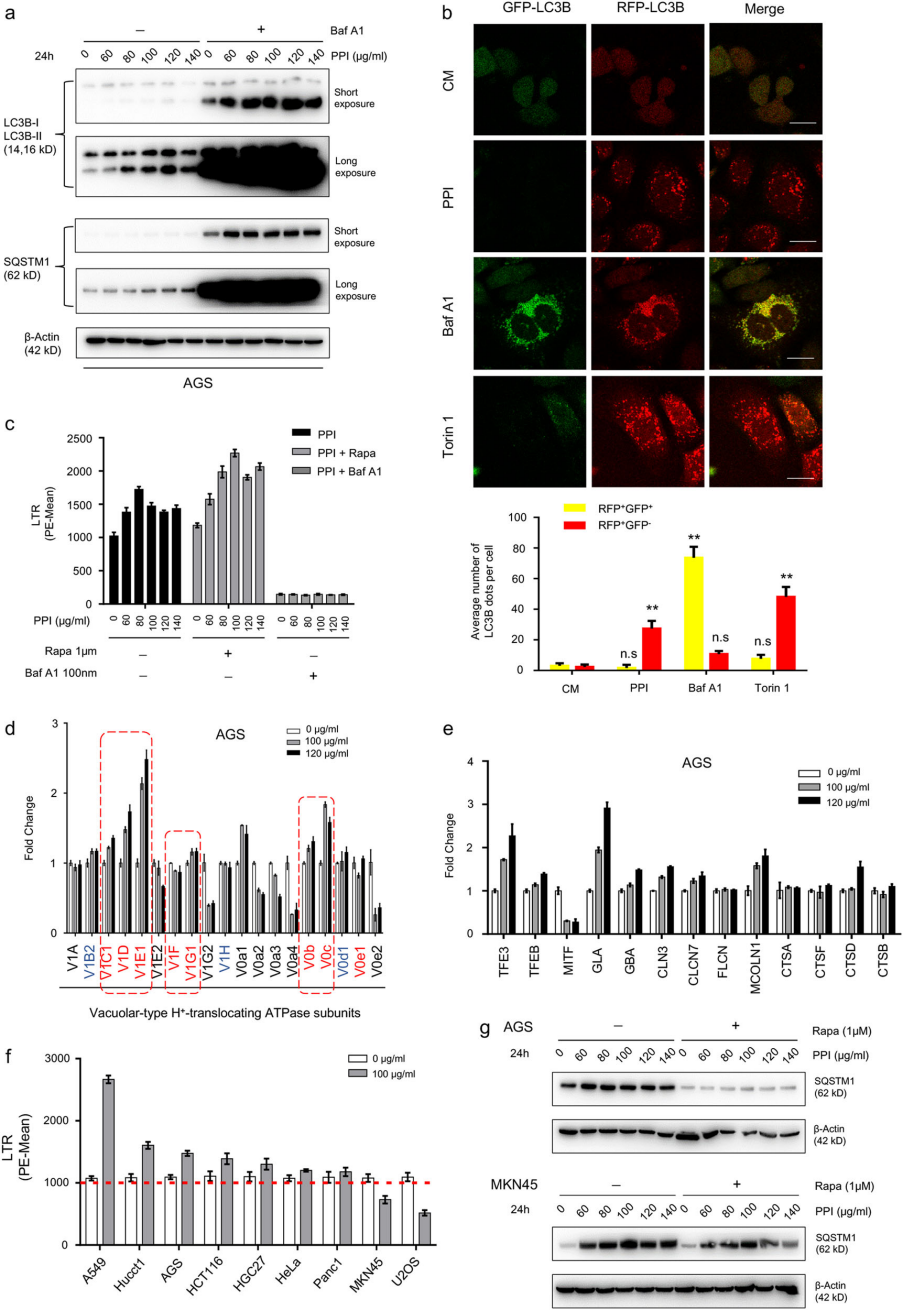
PPI didn’t inhibit the fusion of autophagosomes and lysosomes in neutral pH condition. **a** AGS cells were either untreated or treated with PPI (60–140 μg/ml) for 24 h in pH 7.4 condition in the absence or presence of classical autophagic flux inhibitor baf-A1 (100 nM). The indicated protein levels were analyzed by western blot. **b** AGS cells were transiently infected with GFP-mRFP-LC3B adenoviral particles for 48 h and subsequently treated with PPI (120 μg/ml), baf A1 (100 nM), Torin 1 (500 nM) for 48 h in pH 7.4 condition. The change of both green and red fluorescence was observed using a confocal microscope. Scale bar: 20 μm. Lower panel, the numbers of acidified autophagosomes (GFP^−^RFP^+^) versus neutral autophagosomes (GFP^+^RFP^+^) per cell in each condition were quantified. Data were presented as mean ± SD from three independent experiments (n.s. not significant; **p < 0.01, identified by two-way ANOVA with Dunnett’s multiple comparison test). **c** AGS cells were either untreated or treated with PPI (60–140 μg/ml) for 24 h in pH 7.4 condition in the absence or presence of rapamycin (1 μM) or baf-A1 (100 nM), then stained with LysoTracker Red DND-99 dye (50 nM for 15 min) for FACS analysis. Representative results of three independent experiments were shown. **d**, **e** qRT-PCR analysis of various V-ATPase subunits (**d**) and lysosomal genes (**e**) in AGS cells treated with 100 and 120 μg/ml PPI for 48 h in pH 7.4 condition. Data were presented as mean ± SD. **f** Different cancer cells were either untreated or treated with 100 μg/ml PPI for 48 h in pH 7.4 condition, and then stained with LTR for FACS analysis. Representative results of three independent experiments were shown. **g** AGS and MKN45 cells were pretreated with indicated concentrations of PPI for 24 h in pH 7.4 condition, and then incubated with or without rapamycin (1 μM) for another 24 h. The change pattern of SQSTM1 protein was determined by western blot

Previous works^18,19^ have reported PPI as a V-ATPase inhibitor in low pH condition, with no apparent increase in LC3B and SQSTM1 accumulation after Baf A1 added (Supplementary Figure S4a), increased GFP^+^RFP^+^ puncta (Supplementary Figure S4b) and reduced LysoTracker Red staining (LTR) (Supplementary Figure S4c). This could naturally explain for the accumulation of SQSTM1 after PPI treatment in low pH condition. However, in neutral pH condition, baf A1 dramatically caused further accumulation of LC3B-II by PPI, and PPI still increased the amount of LC3B-II in the presence of baf A1 (Fig. 2a). In line with the change of LC3B-II, dramatic increase in SQSTM1 was also observed (Fig. 2a). In PPI-treated cells, major parts of the LC3B-positive puncta were red, which showed the similar pattern as positive control torin 1 did (Fig. 2b). As shown in Fig. 2c, PPI resulted in a significant increase in acidic vesicles (LTR). In addition, the relatively predominant V-ATPase subunits exhibited an increase after PPI treatment (Fig. 2d and Supplementary Figure S4d-e), albeit to a different degree. Notably, the important regulators of lysosomal biogenesis such as TFE3 and TFEB, accompanied with key lysosomal enzymes, also showed an increased expression (Fig. 2e, Supplementary Figure S5d and S5e). These data indicated that PPI not only promoted autophagosome formation, but also induced lysosomal biogenesis. In order to examine whether the impact of PPI on V-ATPase was merely dependent on the pH condition^18–20^, more PPI-treated cancer cell lines had been screened based on LTR stainning in neutral pH^33^. Most of cell lines tested showed an increased LTR staining, with A549 exibiting the most increment (Fig. 2f and Supplementary Figure S5f), accompanied by increased V-ATPase subunits (Supplementary Figure S5g). However, MKN45 and U2OS showed reduced LTR staining. In MKN45 cells, the accumulation of SQSTM1 was not further increased after baf A1 added (Supplementary Figure S5h), supporting autophagic flux blockage. Unexpectedly, PPI-treated MKN45 cells still showed an increase in V-ATPase subunits (Supplementary Figure S5i), indicating that PPI decreased LTR staining not via reducing V-ATPase subunits, consistent with our findings about PPI in low pH condition. Most important, the PPI-induced SQSTM1 accumulation in neutral pH was sensitive to rapamycin-induced autophagy in AGS cells, while resistent in MKN45 cells (Fig. 2g). Collectively, the impact of PPI on autophagic flux was mainly pH dependent, while regardless of the impact of PPI on LTR staining, PPI-induced lysosome biogenesis in parallel with autophagosome formation.

### The increased SQSTM1 by PPI was transcriptionally mediated by NFE2L2

**Fig. 3:**
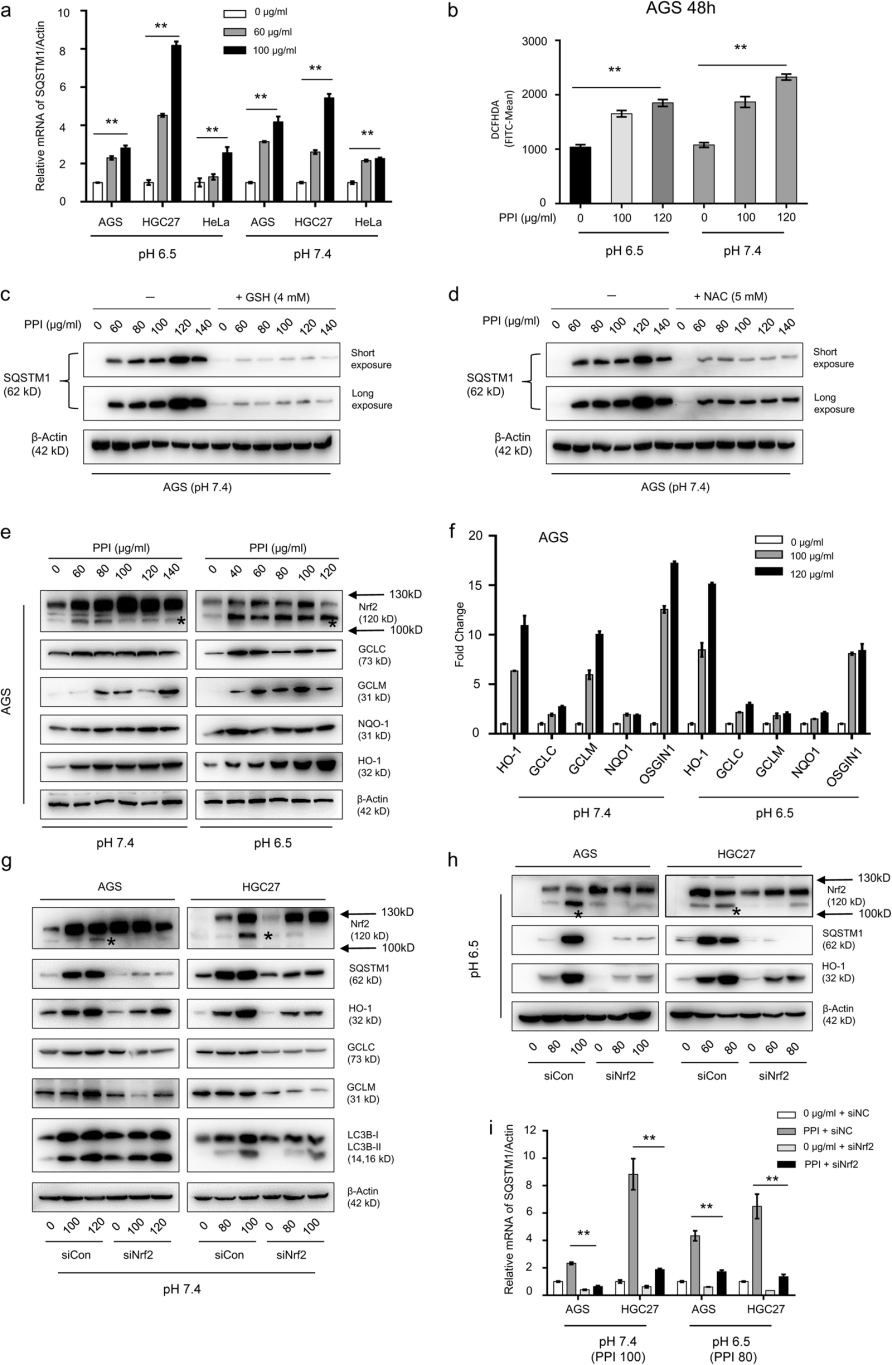
PPI upregulated SQSTM1 mRNA via NFE2L2. **a** AGS, HGC27, and HeLa cells were treated with 60 and 100 μg/ml PPI for 48 h in both pH 6.5 and pH 7.4 conditions, respectively. The SQSTM1 mRNA levels were assessed (**p < 0.01, for each cell line using one-way ANOVA with Dunnett’s multiple comparisons test). **b** AGS cells were treated with PPI for 48 h in both pH 6.5 and pH 7.4 conditions, and then stained with DCFHDA (5 μM) for 30 min. Intracellular ROS was reflected by the fluorescence intensity via flow cytometry analysis. Data presented were representative of three independent experiments (**p < 0.01, identified by one-way ANOVA with Dunnett’s multiple comparisons test). **c**, **d** AGS cells were treated with PPI in the absence or presence of GSH (4 mM) (**c**) or NAC (5 mM) (**d**) for 48 h in pH 7.4 condition. The expression level of SQSTM1 was measured by western blot. **e**, **f** AGS cells were exposed to various concentrations of PPI for 48 h in both pH 6.5 and pH 7.4 conditions. The levels of Nrf2 and its targets such as GCLC, GCLM, NQO1, and HO-1 were assessed by western blot analysis (**e**) and qRT-PCR analysis (**f**). Data were presented as mean ± SD (**p < 0.01, for each gene using one-way ANOVA Dunnett’s multiple comparison test). **g**–**i** AGS and HGC27 cells were reversely transfected with Nrf2 specific siRNA for 48 h, and then exposed to indicated concentrations of PPI for 48 h in both pH 6.5 and pH 7.4 conditions. Knockdown efficiency of Nrf2 was confirmed by western blot analysis (**g**, **h**), and the change pattern of SQSTM1 was evaluated by both western blot (**g**, **h**) and qRT-PCR analysis (**i**). Data were presented as mean ± SD (**p < 0.01, identified by two-way ANOVA with Tukey’s multiple comparisons test)

Since the accumulation of SQSTM1 by PPI was not always a result of autophagic flux blockage such as in neutral pH condition, then we measured the mRNA level of SQSTM1^8^. PPI-induced SQSTM1 mRNA increase occurred both in low and neutral pH conditions, which indicated that this phenomenon was independent of acidic environment (Fig. 3a). Published data showed that SQSTM1 mRNA might be induced by ROS^34^. Cancer cells generated ROS after PPI treatment for 48 h (Fig. 3b and Supplementary Figure S6a) in both conditions as previously reported^18,19^. To clarify the role of ROS in SQSTM1 mRNA increase, we first checked that preincubation with the ROS scavenger GSH (4 mM) or NAC (5 mM) diminished the ROS (data not shown), accompanied with a reversal of cell viability (CCK8), apoptotic phenotype (cleaved PARP) and integrated stress (p-p38) (Supplementary Figure S6b and S6c). PPI-induced SQSTM1 accumulation was significantly abrogated in the presence of NAC or GSH (Fig. 3c, d). Nrf2 is a transcription factor that could be activated in response to ROS. Remarkably, PPI induced Nrf2 expression (Fig. 3e and Supplementary Figure S6d) in both pH conditions, with its increased targets (Fig. 3e, f, Supplementary Figure S6d and S6e)^35^. As there being antioxidant response elements (AREs) in the promoter regions of SQSTM1^36^, Nrf2 could be responsible for increased SQSTM1 mRNA. In Nrf2 deficient cancer cells, the increased SQSTM1 protein and mRNA was reversed (Fig. 3g–i). However, there was no putative AREs in the promoter regions of LC3B^36^. Thus, we failed to detect any significant difference of LC3B-II by PPI between normal and Nrf2-deficient cancer cells (Fig. 3g). The tight relationship between Nrf2 and SQSTM1 was not only tested in vitro, but also confirmed in gastric patients from online databases (TCGA, GSE63089, and GSE27342) (Supplementary Figure S6f). To summarize, these data suggested that Nrf2 activation increased SQSTM1 mRNA in both pH conditions, and contributed to the increased SQSTM1 protein level no matter whether PPI blocked the autophagic flux (pH 6.5) or not (pH 7.4), but was not associated with PPI-induced autophagy.

### Impaired proteasomal degradation also contributed to SQSTM1 accumulation

**Fig. 4:**
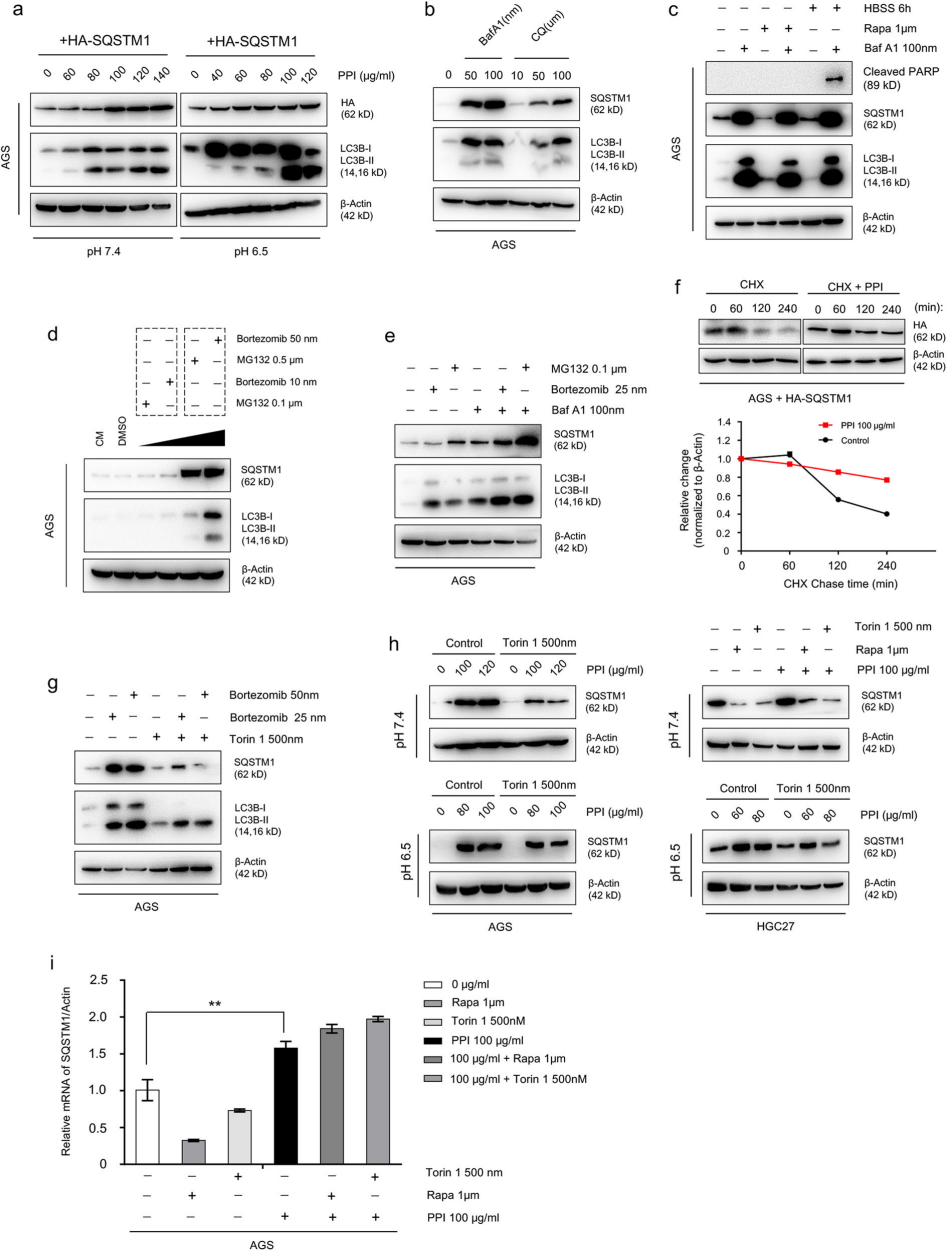
PPI-induced accumulation of exogenous SQSTM1 via ubiquitin-proteasome pathway. **a** AGS cells were transfected with HA-tagged SQSTM1 plasmid for 48 h, and then treated with PPI for another 48 h in both pH 6.5 and pH 7.4 conditions. The exogenous SQSTM1 protein was detected with antibody for HA. **b** AGS cells were treated with baf A1 (50 and 100 nM) and HCQ (10, 50, and 100 μM) for 24 h. **c** AGS cells were pretreated with baf A1 (100 nM) for 30 min, followed with 1 μM rapamycin or amino acid starvation by HBSS for another 24 h. **d** Different concentrations of two classical proteasome inhibitors Bortezomib and MG132 were added. **e** AGS cells were either untreated or treated with Bortezomib (25 nM) or MG132 (0.1 μM) for 24 h in the absence or presence of baf A1 (100 nM). **f** AGS cells transfected with HA-tagged SQSTM1 plasmid, were treated with 25 μg/ml cycloheximide (CHX) over a 240-min time period (left) or treated with 100 μg/ml PPI for 48 h in pH 7.4 conditions, and then followed by 25 μg/ml CHX over a 240-min time period (right). Cells were lysed at the indicated time points (0, 60, 120, and 240 min). Right panel showed the half-life of HA, which reflected the stability of exogenous SQSTM1 protein. **g**–**i** The accumulated SQSTM1 by either proteasome inhibitors or PPI was sensitive to autophagy mediated degradation. AGS cells were pretreated with 25 and 50 nM bortezomib for 1 h, and then incubated with 500 nM torin 1 for 24 h (**g**). After pretreatment with PPI for 24 h in pH 7.4 or pH 6.5 condition, rapamycin (1 μM) or torin 1 (500 nM) was added for another 24 h (**h**). The protein level of SQSTM1 was measured by western blot analysis (**g**, **h**), and the change pattern of SQSTM1 mRNA was confirmed by qRT-PCR (i) (**p < 0.01, identified by one-way ANOVA with Dunnett’s multiple comparison test)

It appeared that Nrf2-mediated transcriptional increase could fully explain SQSTM1 accumulation in neutral pH condition without impaired autophagic degradation^8^. However, to our surprise, in cancer cells overexpressing HA-tagged SQSTM1 plasmid without mRNA interference, PPI increased rather than decreased the levels of HA in neutral pH condition, while it was natural in low pH condition because PPI blocked autophagic flux (Fig. 4a and Supplementary Figure S7). Although SQSTM1 was a common substrate for autophagy, it was also degraded by proteasome and accumulated after proteasome inhibition^2–7^. The SQSTM1 accumulation patterns due to autophagic flux blockage were confirmed (Fig. 4b, c), accompanied with undegraded LC3B-II. Corresponding to autophagy, when UPS inhibited, SQSTM1 also accumulated (Fig. 4d), with a further accumulation upon simultaneous inhibition of autophagy (Fig. 4e). Indeed, PPI treatment prolonged the half-life of exogenous SQSTM1 protein (HA) (Fig. 4f) in neutral pH condition, which indicated that PPI impaired the proteasomal degradation of SQSTM1. It is important to notice that, both in low and neutral pH conditions (Supplementary Figure S8), PPI treatment caused the colocalization of SQSTM1 with Ubiquitin, indicating that SQSTM1 was ubiquitylated, which was the characteristic of proteasome inhibition^37^. More importantly, the accumulated SQSTM1 due to impaired UPS could still be cleared by autophagy (Fig. 4g), indicating no obstructed autophagic flux under proteasome inhibition. Similarly, accumulated SQSTM1 after PPI treatment could also be degraded via autophagy, while in low pH conditions the accumulated SQSTM1 couldn’t be degraded via autophagy because of autophagic flux blockage (Fig. 4h). In order to rule out the interference of mRNA changes, we measured the SQSTM1 mRNA after PPI treatment with or without autophagy inducers. There was no elevated SQSTM1 mRNA reversing back after autophagy activation (Fig. 4i), showing that the decreased SQSTM1 protein level after autophagy activation was not due to reduced transcription. To summarize, the accumulated SQSTM1 was derived from not only increased transcription via Nrf2 but also reduced proteasomal degradation in both pH conditions.

### PPI inhibited proteasome function via reducing proteasome subunits expression

**Fig. 5:**
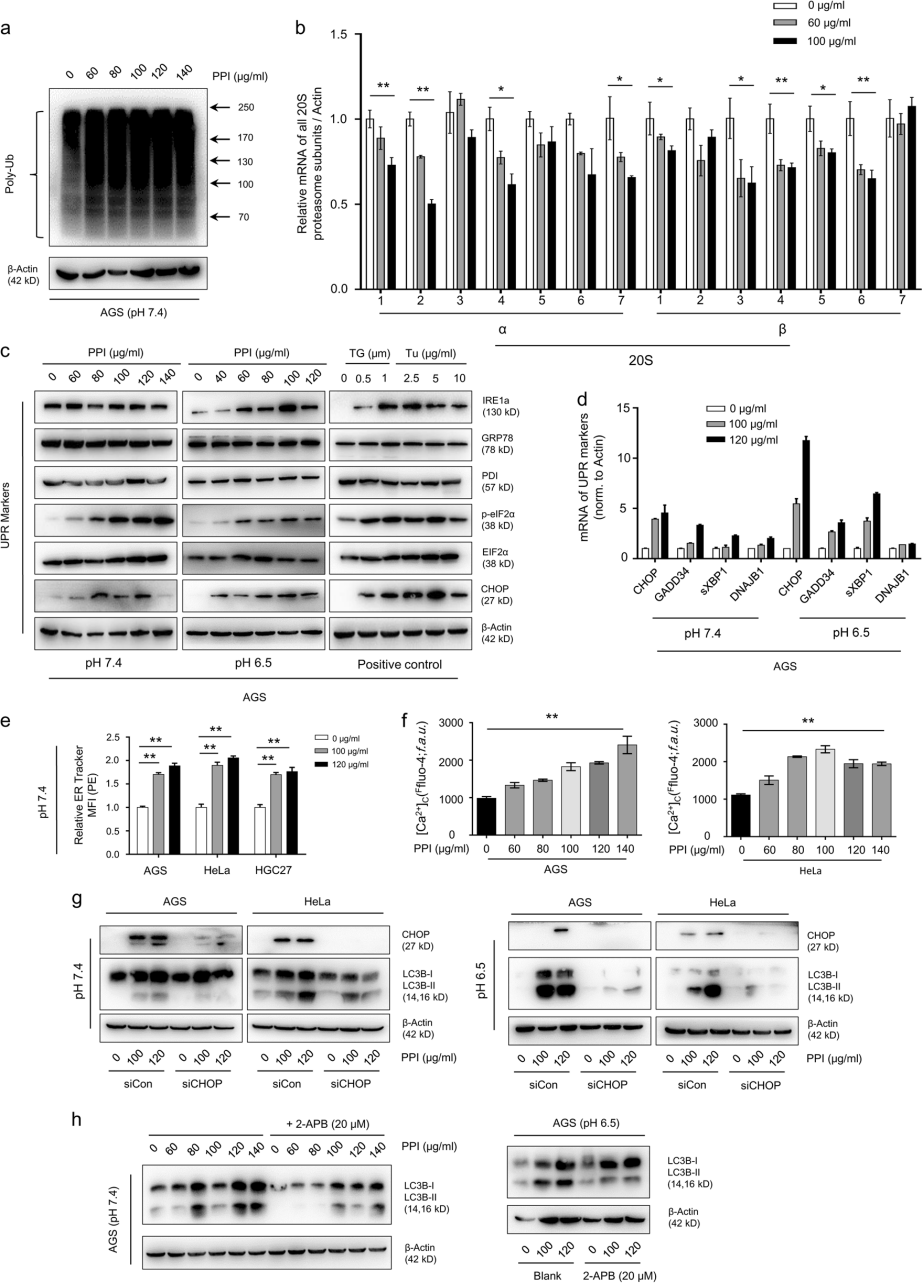
PPI reduced proteasome expression and induced ER stress in cancer cells. **a** AGS cells were treated with indicated concentrations of PPI for 48 h in pH 7.4 condition, followed by measuring proteasome function via western blot analysis using specific antibody to ubiquitin. **b** The mRNA expression level of the 20S proteasome subunits were measured after PPI treatment for 48 h in pH 7.4 condition. Data were presented as mean ± SD (*p < 0.05, **p < 0.01, for each gene using one-way ANOVA with Dunnett’s multiple comparisons test). **c** Western blot analysis of ER stress-related proteins after PPI treatment for 48 h in both pH 7.4 and pH 6.5 conditions. Cells treated with Thapsigargin (TG, 0.5 and 1 μM) or Tunicamycin (Tu, 2.5, 5 and 10 μg/ml) for 24 h served as positive controls. **d** qRT-PCR analysis of UPR genes after 48 h of PPI (100 and 120 μg/ml) treatment in both pH 7.4 and pH 6.5 conditions. Data were presented as mean ± SD (**p < 0.01, for each gene using one-way ANOVA with Dunnett’s multiple comparisons test). **e** ER-Tracker Red (500 nM) staining of PPI treated cells in pH 7.4 condition was performed. The quantification of ER Tracker fluorescence were accomplished by FACS. Data presented were representative of three independent experiments (**p < 0.01, for each cell line using one-way ANOVA with Dunnett’s multiple comparisons test). **f** After treated with PPI (60–140 μg/ml) for 24 h in pH 7.4 condition, cells were then incubated with 5 μM Fluo-4/AM and detected by flow cytometry. Data presented were representative of three independent experiments (**p < 0.01, identified by one-way ANOVA with Dunnett’s multiple comparisons test). **g**, **h** PPI-induced ER stress contributed to the acitivation of autophagy. AGS and HeLa cells transfected with CHOP-specific siRNA, were treated with 100 and 120 μg/ml PPI for 24 h in both pH 7.4 and pH 6.5 conditions (**g**). AGS cells were treated with the indicated concentrations of PPI in the absence or presence of 2-APB (20 μM) for 24 h in both pH 7.4 and pH 6.5 conditions (**h**). The autophagy marker LC3B-II was determined

According to the results above, we considered the UPS as a new potential target of PPI. First of all, poly-ubiquitinated proteins increased (Fig. 5a and Supplementary Figure S9a), suggesting that PPI inhibited proteasome function. TP53, the short-lived protein degraded by proteasome^2,6^, accumulated after PPI treatment (Supplementary Figure S9b). Secondly, due to lacking the aromatic ketone structures^38^, PPI might inhibit the proteasome function on the transcriptional level. We noticed that PPI affected the mRNA of several proteasome and immunoproteasome subunits with a downward trend (Fig. 5b and Supplementary Figure S9c), which contributed to proteasome activity^39,40^. Prior work revealed a potential link between STAT3 and its transcriptional control of immunoproteasome subunits^41^. Along with the inhibited transcriptional activity of STAT3 (Supplementary Figure S10a)^42^, PPI reduced mRNA expression of PSMB8 and PSMB9 (Supplementary Figure S10b) in both pH conditions. We also confirmed the mRNA correlation between STAT3, PSMB8 and PSMB9 in online databases (Supplementary Figure S10c). However, the definite STAT3 binding sites were only confirmed in the promoter regions of PSMB8 and PSMB9^41^, while PPI could make other proteasome subunits downregulated. As a result, PPI maybe reduce proteasome subunits expression via far more than just inhibiting STAT3.

### ER stress/UPR was initiated upon proteasome inhibition by PPI

An inactive proteasome made unfolded proteins accumulated in the ER, and then caused ER stress/UPR. We screened for ER stress related markers, with CHOP and p-eIF2a (Ser 52) showing a dose-dependent increase (Fig. 5c, d, Supplementary Figure S11a and S11b). ER Tracker staining and quantification by flow cytometry indicated ER expansion in PPI-treated cells (Fig. 5e, Supplementary Figure S11c)^43^. In addition, perturbations in Ca^2+^ homeostasis upon ER stress were observed as early as for 24 h (Fig. 5f and Supplementary Figure S11d). The mRNA expression of SERCA (Sarco/ER Ca^2+^-ATPase for influx) and IP3R (inositol trisphosphate (IP3) receptor for efflux) was also examined, revealing a decrease in SERCA (the target of thapsigargin (TG)) and an increase in the most prominent isoform, ITPR3 (Supplementary Figure S11e and S11f). Protein overload in the ER induces autophagy^44,45^. Both CHOP siRNA and the IP3Rs inhibitor 2-APB, could partially reverse the induction of LC3B-II by PPI (Fig. 5g, h) in both pH conditions, suggesting that ER stress after proteasome inhibition was located upstream of PPI-induced autophagy independent of pH conditions.

### PPI sensitized cancer cells to protein overload stress

**Fig. 6:**
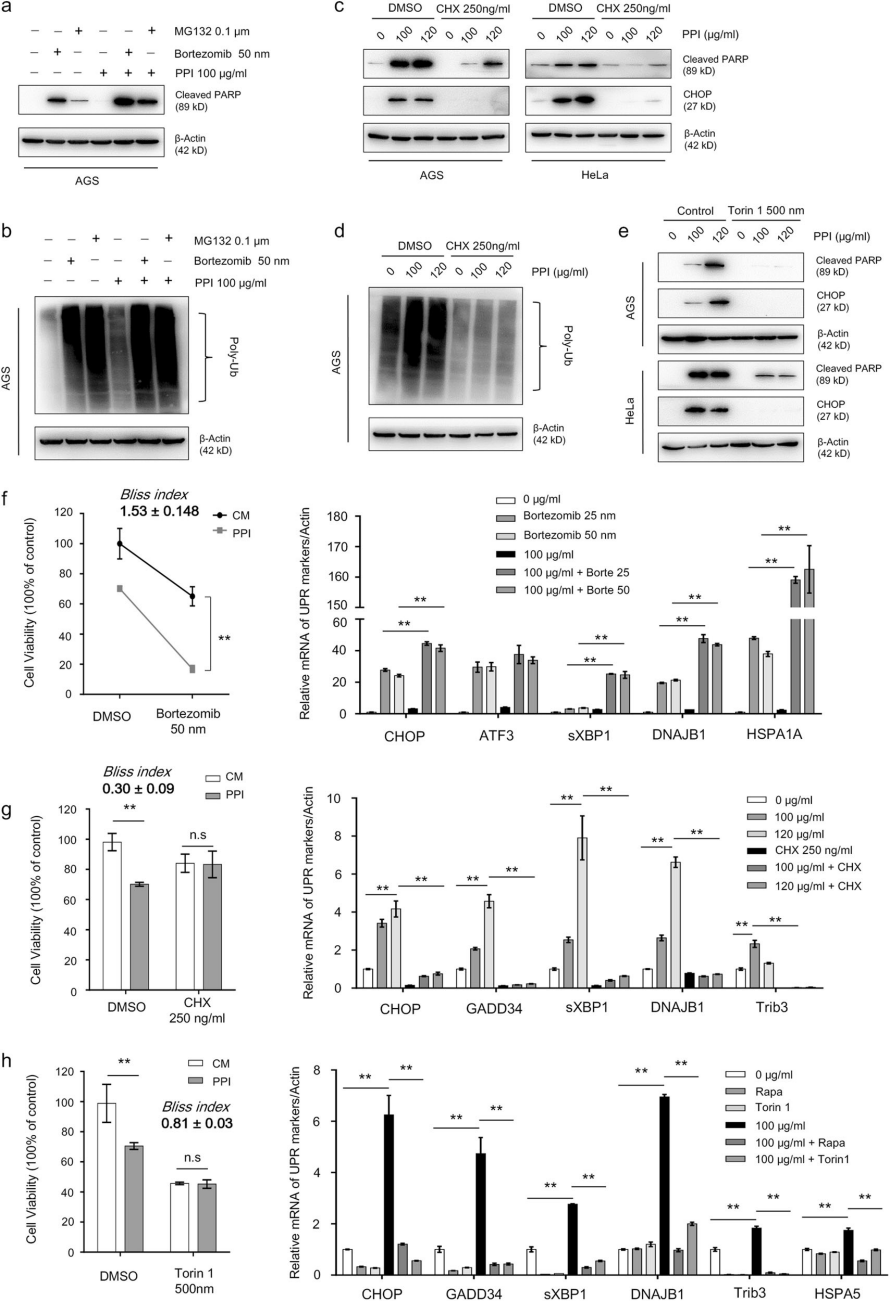
Proteasome inhibitors aggravated while protein synthesis suppression ameliorated the UPR and cell death by PPI. **a**, **b** AGS cells were pretreated with PPI (100 μg/ml) for 24 h in pH 7.4 condition, followed by combination with or without 50 nM Bortezomib or 0.1 μM MG132 for another 24 h. Western blot analysis of apoptosis related protein cleaved-PARP levels (**a**) and poly-ubiquitinated proteins (**b**). **c**, **d** AGS and HeLa cells were pretreated with CHX (250 ng/ml) for 2 h, and then incubated with PPI (100 μg/ml or 120 μg/ml) for 48 h in pH 7.4 condition. Levels of apoptosis related protein cleaved-PARP, UPR marker CHOP (**c**), and poly-ubiquitinated proteins (**d**) were analyzed. **e** AGS and HeLa cells were pretreated with torin 1 (500 nM) for 2 h, and then incubated with PPI (100 μg/ml or 120 μg/ml) for 48 h in pH 7.4 condition. Indicated proteins were analyzed by western blot. **f** AGS cells were treated as described in (**a**). The cell viability was determined by CCK8 assay (left) (**p < 0.01, identified by two-way ANOVA with Sidak’s multiple comparisons test). qRT-PCR analysis of UPR genes was performed (right) (**p < 0.01, for each gene using one-way ANOVA with Tukey’s multiple comparisons test). **g** AGS cells were treated as described in (**c**). The cell viability was determined by CCK8 assay (left) (**p < 0.01, identified by two-way ANOVA with Sidak’s multiple comparisons test). qRT-PCR analysis of UPR genes was performed (right) (**p < 0.01, for each gene using one-way ANOVA with Tukey’s multiple comparisons test). **h** AGS cells were treated as described in (**e**). The cell viability was determined by CCK8 assay (left) (**p < 0.01, identified by two-way ANOVA with Sidak’s multiple comparisons test). qRT-PCR analysis of UPR genes was performed (right) (**p < 0.01, for each gene using one-way ANOVA with Tukey’s multiple comparisons test)

Sustained proteasome inhibition could lead to uncontrolled UPR and subsequent cell death. After pretreated with PPI for 24 h, more AGS cells underwent apoptosis (Fig. 6a) when proteasome inhibition was aggravated by bortezomib or MG132, along with more poly-ubiquitinated proteins accumulated (Fig. 6b). Cell viability assay showed that, in the presence of bortezomib, PPI displayed a more profound cytotoxic effect, confirmed by Bliss index (1.53 ± 0.148) (pH 7.4) (Fig. 6f) and Bliss index (1.34 ± 0.115) (pH 6.5) (Supplementary Figure S12a). As a marker for protein overload stress^43^, the UPR genes also showed a further induction in combined approach. On the other side, preventing protein synthesis by cycloheximide (CHX) could lessen PPI-induced UPR and apoptosis (Fig. 6c) and poly-ubiquitinated proteins accumulation (Fig. 6d). Reducing protein synthesis by mTOR inhibitor rapamycin or torin 1 also achieved similar results (Fig. 6e). In cells with low levels of protein synthesis, the cytotoxic effect of PPI decreased, with both Bliss indexs (pH 7.4: CHX: 0.30 ± 0.09; Torin 1: 0.81 ± 0.03. pH 6.5: CHX: 0.75 ± 0.15; Torin 1: 0.77 ± 0.08) lower than 1, and the increased UPR genes reverting to normal levels (Fig. 6g, h, and Supplementary Figure S12b and S12c).

Autophagy is often activated as an important compensatory response for proteasome inhibitors^4–6,46^. Further inhibiting proteasome by bortezomib promoted PPI-induced autophagy (Supplementary Figure S13a and S13b). On the contrary, when protein synthesis was aborgated or suppressed, the induced autophagy by PPI was reverted (Supplementary Figure S13c-f). Simultaneous inhibition of autophagy upon bortezomib treatment caused further accumulation of ubiquitinated proteins, and made cells more prone to apoptosis (Supplementary Figure S14a and S14b). Similarly, when the early stage of autophagy was genetically inhibited, PPI also made more cancer cells undergo apoptosis (Supplementary Figure S14c), even though the degree of increase varied in cell lines. When autophagic flux was inhibited, consistent results were achieved (Supplementary Figure S14d and S14e). Given the fact that PPI blocked autophagic flux in low pH condition, there was an interesting comparison showing that, the same amount of PPI caused more protein overload stress and UPR markers in pH 6.5 than pH 7.4, which was quantified by ER Tracker staining (Supplementary Figure S14f) and Q-PCR (Supplementary Figure S14g). Similar results were also achieved by adding HCQ into the PPI-treated cancer cells in pH 7.4, which indicating that the increased protein overload stress in pH 6.5 was due to autophagy inhibition.

These observations suggested that, based on the proteasome inhibitory effect of PPI, both proteasome and autophagy inhibitors would have a synergistic interaction with PPI, and PPI sensitized cancer cells to death under protein overload stress.

### The Bcl-2 inhibitors synergized the cytotoxicity of PPI

**Fig. 7:**
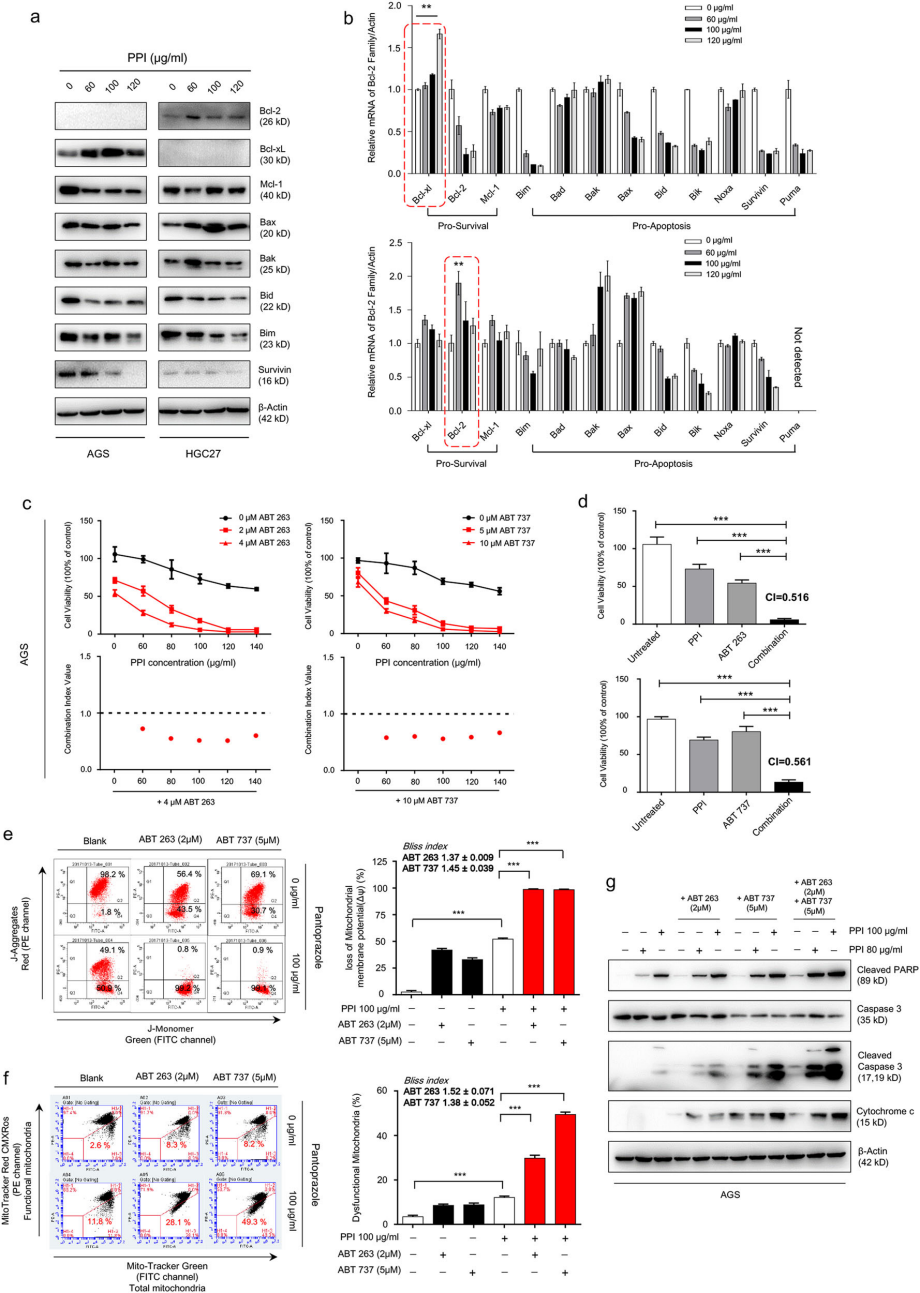
Bcl-2 inhibitors synergized the cytotoxicity of PPI. **a**, **b** The Bcl-2 family members were analyzed by western blot (**a**) and qRT-PCR (**b**). Data were presented as mean ± SD (**p < 0.01, for each gene using one-way ANOVA with Dunnett’s multiple comparisons test). **c** AGS cells were pretreated with various concentrations of PPI for 24 h in pH 7.4 condition, and then incubated with two different doses of ABT-263/ABT-737 for another 24 h. Data were presented as percentages of cell viability as determined by CCK8 assays (upper panel). Synergisms of cell viability inhibition by the combination therapy were analyzed by Combination Index Value (lower panel). **d** Cell viability of AGS cells after treatment with combination of 100 μg/ml PPI and 4 μM ABT-263/10 μM ABT-737 in pH 7.4 condition. The combination indexes (CI) were calculated as described in Methods section. Data were presented as mean ± SD (***p < 0.001, indentified by one-way ANOVA Dunnett’s multiple comparison test). **e** AGS cells were treated as described in (**c**), and the changes in mitochondrial membrane potential (Δψm) were analyzed by JC-1 assay. Ratio of green to red fluorescence was depicted (left panel). Data were representative of three independent experiments. Right panel showed the quantification of the Δψm in AGS cells upon combinational treatment. Data were presented as mean ± SD (***p < 0.001, indentified by one-way ANOVA Tukey’s multiple comparisons test). **f** AGS cells were treated as described in (**c**), and then stained with MitoTracker green (100 nM) and MitoTracker red (400 nM). The dysfunctional mitochondria accumulation was analyzed by FACS. Dot plots of subpopulation are depicted (left panel) and percent of dysfunctional mitochondria were shown as mean ± SD (right panel) (***p < 0.001, indentified by one-way ANOVA Tukey’s multiple comparisons test). **g** AGS cells were treated as described in (**c**). The apoptosis related proteins were detected

Similar to previous results^18,23,24^, in gastric cancer cells, caspase activation is involved in PPI-induced cell death, but caspase inhibition could only partially reduce PPI-induced cell death in both conditions (data not shown). Taking into account the increased mROS (Supplementary Figure S15a) and influx of calcium into mitochondria (Supplementary Figure S15b) after PPI treatment, cotargeting mitochondria together with PPI may represent a new platform to sensitize cancer cells to death. Apoptosis was guarded by Bcl-2 family proteins^12^. We found the interesting trend of antiapoptotic proteins increasing and proapoptotic proteins decreasing (Fig. 7a, b), which may limit the pro-cell death effect of PPI. We pretreated PPI for 24 h and then added ABT-263/737 for another 24 h. This combination therapy produced a significantly advantage over each drug alone (Fig. 7c, Supplementary Figure S15c, Supplementary Figure S16a and S16c). Formal synergy analyses were performed, and the synergistic cell viability inhibitory effect was observed (Fig. 7c, d, Supplementary Figure S15c and S15d, Supplementary Figure S16c and S16d). The increased cell death was also confirmed (Supplementary Figure S15e and S15f), and the combination strategy almost totally induced mitochondrial membrane depolarization (Fig. 7e and Supplementary Figure S15f). Correspondly, more damage was brought to mitochondria (Fig. 7f)^47^. The synergistic cytotoxicity of combination therapy was also indicated by increased apoptosis with elevated cleaved PARP and cleaved caspase 3, accompanied with more cytochrome c releasing into cytoplasm (Fig. 7g). These data provided support that, cotargeting induced antiapoptotic Bcl-2 proteins by PPI, was an important complementary strategy for strengthening the cytotoxicity of PPI, both in low pH and neutral pH conditions.

## Discussion

Previous works have shown that the accumulation of LC3B and SQSTM1 by PPI in low pH condition, was associated with the inhibitory effect of PPI on V-ATPase. However, according to our work, in neural pH condition where PPI was unable to inhibit V-ATPase, PPI could still cause LC3B and SQSTM1 accumulation. These results showed that the impacts of PPI on autophagy were not merely the autophagic flux blockage in low pH condition^19^. Recent studies also found that TM9SF4 could regulate autophagy initiation in response to nutrient starvation by inhibiting the nutrient-sensing kinase complex mTORC1^30^. Indeed, we found that PPI markedly reduced the mTOR activity consistent with previous reports^25^, as indicated by dephosphorylation of mTOR, 4E-BP1 and RPS6. Intriguingly, TM9SF4-siRNAs substantially attenuated the PPI-induced mTOR inactivation, and more important LC3B accumulation. These data provide strong evidence that TM9SF4 acts through mTOR to facilitate autophagosomes formation induced by PPI in different pH conditions.

**Fig. 8:**
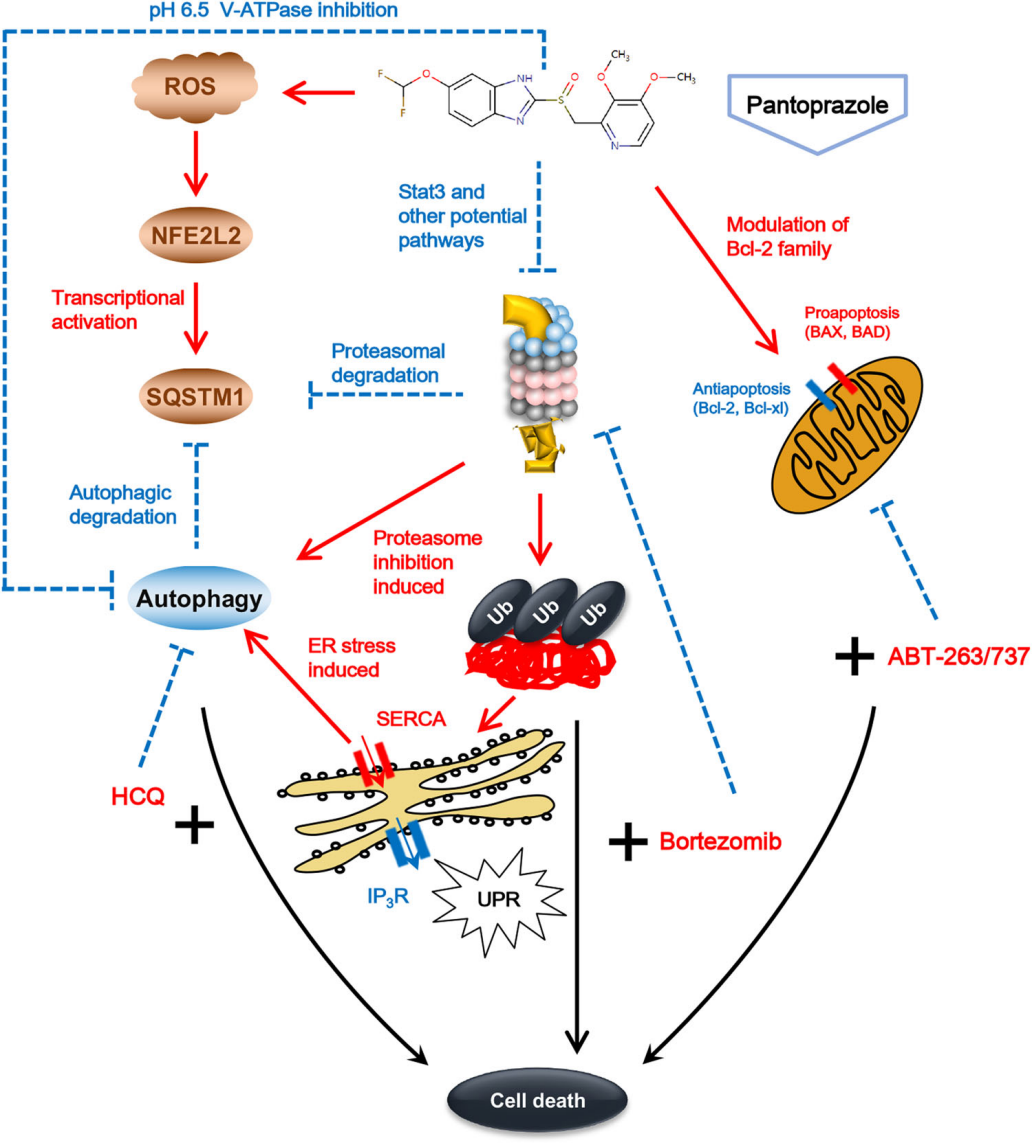
Schematic illustration of proposed mechanisms. PPI inhibited proteasome function, and induced SQSTM1 elevated autophagy as a compensatory response for impaired proteasomal degradation. PPI promotes autophagic flux in neutral pH condition while blocks autophagic flux in low pH condition. When cancer cells were under proteins overload stress caused by proteasome inhibitors and autophagic flux blockers, or mitochondrial stress caused by Bcl-2 inhibitors, the cytotoxicity of PPI would be significantly increased

SQSTM1 accumulation in neutral pH condition after PPI treatment without V-ATPase inhibition also made the actual sources of increased SQSTM1 confusing^9^. Consistent with the increased SQSTM1 mRNA in esomeprazole-treated EAC cells^22^, PPI elevated SQSTM1 mRNA via Nrf2 in both pH conditions dependent on ROS generation^35,36^. Our inertial thinking usually assumed SQSTM1 accumulation to be totally mediated by Nrf2 especially in neutral pH condition, but the accumulation of exogenously expressed SQSTM1 made us aware of the potential decreased protein degradation by other system, such as UPS. In our hands, PPI inhibited proteasome function via reducing various proteasome subunits expression, even though the responsible transcription factors besides STAT3 must be elucidated in future. The colocalization of SQSTM1 with Ub indicated that, proteasome function was inhibited after PPI treatment, and then SQSTM1 was ubiquitylated and mediated the transient aggregation of ubiquitinated proteins, such as proteasome, aggresome, and ALIS formation to regulate the proteostasis^7^. The specific progress mediated and structure formed by SQSTM1 after PPI treatment needs further investigations.

Proteostasis is a key requirement for cancer cell viability and stress adaptation, with protein degradation systems maintaining the balance^2^. Our results indicated that heightened protein synthesis was a prominent source of ER stress/UPR in PPI treated cancer cells. Further inhibiting proteasome function with bortezomib augmented the PPI-induced UPR, and triggered synergistic cytotoxicity compared with either drug alone. Autophagy was activated to counterbalance ER expansion during UPR^44,45^. Indeed, we found that knockdown CHOP or inhibiting ER Ca^2+^ efflux by 2-APB markedly reduced the accumulation of LC3B. Since autophagy plays a role in loosening protein overload stress, cotargeting autophagy may be a promising approach for amplifying the tumor killing effects of proteasome inhibitors^48–50^. Notably, in our hands, autophagy inhibition with HCQ or Atg5/Atg7 RNAi sensitized cancer cells to PPI-induced cell death. Moreover, compared with the cells in neutral pH condition, cells in low pH condition showed more protein overload stress, accompanied with increased apoptosis (data not shown). These results presented herein are in keeping with previous reports supporting a protective role of PPI-induced autophagy^19^. To summarize, PPI treatment could sensitize cancer cells to death under protein overload stress either by proteasome inhibition or autophagy deficiency, and the combination strategies of PPI with both proteasome inhibitors and autophagic flux blockers would be promising approaches for cancer treatment (Fig. 8).

Bcl-2 inhibitors targeting against antiapoptotic family members offer the prospect of silencing prosurvival pathways^12^, and lower the threshold required to initialize apoptosis. Considering for the induced antiapoptotic proteins after PPI treatment, the cytotoxicity of PPI significantly increased when combined with Bcl-2 inhibitors. The pro-cell death effect of PPI was correlated with, or may be partially governed by, the proximity of cancer cell mitochondria to the apoptotic threshold, which could be shortened by Bcl-2 inhibitors. Future studies that characterize the specific pathways mediating synthetic lethality following PPI and Bcl-2 inhibitors may provide additional strategies to enhance cancer cell death.

## Materials and methods

### Cell culture

The human cancer cell lines including AGS, HGC27, HeLa, Panc-1, HCT116, A549, and U2OS were purchased from the Cell Resource Center, Institute of Biochemistry and Cell Biology, Shanghai Institutes for Biological Sciences, Chinese Academy of Sciences (Shanghai, China). MKN45 was purchased from the Cell Resource Center, Institute of Basic Medical Sciences, Chinese Academy of Medical Sciences (Beijing, China). Hucct1 were obtained from the Japanese Collection of Research Bioresources (JCRB) (Tokyo, Japan). The cell lines were originally authenticated in China Center for Type Culture Collection (CCTCC) by Short Tandem Repeat (STR) profiling and passaged less than 6 months in the lab. These cells are routinely maintained in the laboratory of our group.The acid cell culture medium (pH 6.5) was obtained by the addition of 1 M HCl solution as previously reported^23,24^. The pH was estimated by the use of a FE20 FiveEasy Plus pH Meter (METTLER TOLEDO Instruments (China), Shanghai, China).

### Cell viability assay and quantitative methods for drug interaction analysis

Different cancer cells (5000 cells per well) were seeded into 96-well plates and then allowed for adherence and culture overnight at 37 ℃. PPI was dissolved in complete medium (CM), and other different reagents were dissolved in optimal solvent such as DMSO, whose final concentration was guaranteed to be less than 1‰. The order in which the drugs were added, and the time of sustained exposure, were described in detail in the section of Results and Figure legends. Hundred microliter CCK-8 working solutions were added into each well of 96-well plates after treatment, and plates were incubated at 37 ℃ for another 1–1.5 h. The absorbance at 450 nm was measured, and the percentage of cell viability or cell viability inhibition was calculated by using the absorbance values of untreated control cells (C), and drug-treated cells (T) as follows: T/C × 100 for cell viability, where untreated cells set as 100%, and (C–T)/C × 100 for cell viability inhibition, where untreated cells set as 0%. All experiments were carried out in quintuplicate for technical repetition, and performed in triplicate for biologic repetition.

To assess the synthetical effect of pantoprazole with other drugs, we adopted two methods for determining the drug interaction under different circumstances. One is to calculate the ratio of observed [Eobs(*x*,*y*)] and expected [Eexp(*x*,*y*)] responses according to the Bliss method^51^. The formula for predicting expected effect is as follows: Eexp(*x*,*y*) = Ex + Ey−(Ex*Ey), where Ex and Ey are each separate drug effect. It should be noted that the Bliss index of 1, <1 or >1 indicates additive, antagonistic or synergistic effects, respectively. CI is an another quantitative measure for determing the degree of drug interaction with synergism (CI< 1), additive effect (CI = 1), or antagonism (CI > 1) for a given final measurement, which is different from the Bliss index^52^. CIs were calculated by using CalcuSyn software program (BIOSOFT, Cambridge, UK).

### Reagents and drugs

Pantoprazole sodium in powder (PANTOLOC) was purchased from Takeda GmbH (Germany). Bafilomycin A1 (Baf-A1, S1413), Hydroxychloroquine Sulfate (HCQ, S4430), Rapamycin (Rapa, S1039), Wortmannin (WM, S2758), and Nutlin-3a (S8059), were purchased from Selleck Chemicals (Houston, TX, USA). N-acetyl-L-cysteine (NAC, A7250), 2′,7′-Dichlorofluorescin diacetate (DCFHDA, D6883), L-Glutathione reduced (GSH, G4251), Thapsigargin (TG, T9033), and 2-Aminoethyl diphenylborinate (2-APB, D9754) were purchased from Sigma-Aldrich (St. Louis, MO, USA). Torin 1 (HY-13003), Tunicamycin (Tu, HY-A0098), Cycloheximide (CHX, HY-12320), Bortezomib (borte, HY-10227), and MG-132 (HY-13259) were purchased from MedChem Express (Shanghai, China). LysoTracker Red DND-99 (L7528), and Fluo-4,AM (F23917), Rhod-2,AM (R1245MP), MitoSOX™ Red (M36008), MitoTracker™ Green (M7514), MitoTracker™ Red CMXRos (M7512), and JC-1 Mitochondrial Membrane Potential Dye (T3168) were purchased from Molecular Probes (USA). ABT-263 (Navitoclax) (A3007) and ABT-737 (A8193) were purchased from Apex Bio (Houston, TX, USA). Lipofectamine 3000 (L3000008) and Lipofectamine RNAiMAX (13778150) were purchased from Invitrogen.

### Plasmids, siRNAs and transfections

The pBABE-puro-EGFP-LC3 was purchased from Addgene (22405, deposited by Jayanta Debnath (University of California, San Francisco, CA, USA)). Full-length HA-tagged SQSTM1 and HA-tagged Ubiquitin plasmids were kind gifts from Dr. Mingming Zhang (Nanjing, China). For plasmids transfection, AGS and HeLa cells were seeded into 6-well plates at a density of 20–25 × 10^4^ cells/well for overnight and transiently transfected using Lipofectamine 3000 according to the manufacturer’s instructions. The siRNA oligonucleotides targeting Atg5, Atg7, Beclin 1, Nrf2, CHOP, and negative control siRNA were all chemically synthesized by RiboBio (Guangzhou, China) and resuspended in RNase-free water to a stock concentration of 20 μM. The siRNA sequences were detailed in Supplementary Table 1. AGS, HGC27, and HeLa cells were reversely transfected as follows: 5 μl of 20 μM siRNA and 5 μl Lipofectamine RNAiMAX were mixed with 400 μl of Opti-MEM (Gibco) in sterilized 1.5 ml EP tubes. 1.6 ml of cell suspension including 30 × 10^4^ cells in complete growth medium without antibiotics was first added into each well of six-well plate. After incubation for 20 min at room temperature, the siRNA-lipid complex was then added gently into cells in a spiral pattern. This gave a final siRNA working concentration of 50 nM.

### Western blot analysis and antibodies

Cells were lysed in RIPA buffer (Beyotime Biotechnology, P0013B) containing EDTA-free protease inhibitor cocktail (Roche, 04693159001) on ice for 30 min, and then centrifuged for 15 min (13,000 × *g*, 4 ℃). The supernatant was collected, and protein concentrations were determined by BCA Protein Assay Kit (KeyGEN BioTECH, KGP903). Equal amounts of protein (at least 30 μg) were separated on 8–12% SDS-PAGE and then electrophoretically transferred onto a PVDF membrane (Millipore, Bedford, MA, USA). After blocking with 5% nonfat milk in Tris-buffered saline containing 0.1% Tween-20 for 2–4 h, the membranes were incubated with specific primary antibodies according to the instructions overnight at 4 ℃, followed by incubation with appropriate horseradish peroxidase-conjugated secondary antibodies (1:3000 dilutions) for 2 h. Signals generated by enhanced chemiluminescence (Millipore) were recorded by Tanon 4600 imaging system (Tanon, Shanghai, China). Data are representative of at least three independent experiments.

All primary antibodies used in this study were diluted according to the instructions. The catalogue numbers and manufacturers were described in Supplementary Table 2.

### Transmission electron microscopy

Cells were seeded in 75 cm² cell culture flasks (Corning, 430641) at 5 × 10^6^ cells/flask. PPI (120 μg/ml) was added to the flasks on the following day. After 48 h treatment, cells were collected and fixed with 2.5% glutaraldehyde in 0.1 M sodium dihydrogen phosphate (pH 7.4). The samples were then fixed with 1% OsO_4_ in 0.1 M cacodylate buffer (pH 7.2) containing 0.1% CaCl_2_ for 1 h, dehydrated through a graded series of ethanol (30–100%), and gradually infiltrated with epoxy resin (GENMED, GMS11012). Ultra-thin sections were obtained and stained with 4% uranyl acetate and lead citrate, and examined in a transmission electron microscope (JEM-200CX, JEOL, Japan).

### Immunofluorescence

The pBABE-puro-EGFP-LC3 plasmid was transiently transfected in AGS and HeLa cells. After 48 h of transfection, the cells underwent designated treatments for further experiments. The GFP-LC3B dots were observed and captured under cellSens fluorescence microscope (Olympus, Tokyo, Japan). The average number of GFP-LC3B dots per cell was determined from three independent experiments. AGS cells were grown on collagen-precoated glass coverslips directly in 24-well plates. After appropriate treatments, cells were fixed with 4% paraformaldehyde, permeabilized with 0.1% Triton X-100, blocked with 10% normal goat serum for 1 h, incubated with indicated primary antibodies and subsequently corresponding Alexa Fluor-labeled secondary antibodies, mounted using the Prolong Gold Antifade reagent with DAPI (Sigma), and viewed using a cellSens fluorescence microscope.

### Live-cell imaging for autophagic flux

The mRFP-GFP-LC3 adenoviral particles were purchased from HanBio (Shanghai, China). Cells (10 × 10^4^ cells per well) were seeded into 6-well plates and then allowed for adherence overnight at 37 ℃. Then cells were infected with adenoviral particles at a MOI of 100 in complete medium without antibiotics; after infection for 24 h, the medium was replaced with fresh medium with antibiotics, and the cells were cultured for another 24 h before various treatments. Imaging was performed on a Leica SP8 Confocal System (Carl Zeiss, Germany). All image acquisition settings were kept same.

### Quantitative real-time PCR with reverse transcription (qRT–PCR)

After designated treatments, total RNA was isolated from cells using RNAiso Plus Reagent (Takara, 9109). Approximately 1000 ng RNA was reversely transcribed into cDNAs using 5 × PrimeScript RT Master Mix (Takara, RR036A) in a 10 μl reaction mixture. The reaction conditions were: 37 ℃ for 15 min, 85 ℃ for 5 s. Quantification of gene expression was performed using the SYBR Premix Ex TaqTM II kit (TaKaRa, Kyoto, Japan) on the LightCycler 96 system (Roche, Mannheim, Germany). The reaction conditions were: 95 ℃ for 10 min, followed by 40 cycles of 95 ℃ for 15 s and 60 ℃ for 1 min. All reactions were performed in triplicate. Primers used in real-time PCR experiments were shown in Supplementary Table 3.

### Detection of cell death

Cells were seeded in a six-well plate at a density of 30 × 10^4^ cells per well, and then exposed to appropriate treatments for indicated times. The cell death was quantitatively and qualitatively analyzed by the following approaches: (1) PI staining (BD Biosciences, 556463) assay by FACS according to the manufacturer’s protocol; (2) the level of PARP and caspase-3 cleavages were detected by western blotting. Both the floating and adherent cells were collected, and then washed twice with phosphate buffered saline (PBS). After harvested, cells were immediately resuspended in 300 μl PBS buffer followed by incubation with propidium iodide (PI; final concentration of 5 μg/ml) for 10 min. The percentage of PI-positive cells, in the name of dead cells, was quantified by BD Accuri™ C6 (BD, Becton, Dickinson, USA).

### JC-1 assay

AGS and HGC27 cells were pretreated with PPI for 24 h, and then incubated with or without Bcl-2 inhibitors (ABT-263 or ABT-737) for another 24 h. Adherent cells were digested by trypsin and collected with floating cells. Harvested cells was washed twice with PBS, and then incubated with JC-1 dye for 25 min at 37 °C. The cells with loss of mitochondrial membrane potential (Δψm), which showed decreased red fluorescent J-aggregates, were detected by BD FACSAria II (BD, Becton, Dickinson, USA). Data were analyzed by FlowJo software (Version 7.6.5, Tree Star Inc., Ashland, Oregon).

### LysoTracker Red staining

After the planned treatments, cells were harvested and incubated with 50 nM LysoTracker Red DND-99 (LTR, L7528) in PBS for 30 min to label acidic organelles in live cells. After twice washing, cells were suspended in PBS and transferred into 1.5 ml EP tubes. The fluorescence intensities of at least 10,000 cells/sample were measured by BD FACSAria II (BD, Becton, Dickinson, USA).

### Dysfunctional mitochondria labeling

For labeling mitochondria, the cells were incubated with 100 nM MitoTracker Green FM (total mitochondria) and 400 nM MitoTracker Red CMXRos (functional mitochondria) for 25 min at 37 °C. The labelled cells were then washed twice with PBS followed by resuspended in PBS for flow cytometry. The subpopulation was distinguished by BD Accuri™ C6 (BD, Becton, Dickinson, USA). Dysfunctional mitochondria was reflected by decreased red fluorescence intensity under the same green fluorescence intensity^47^.

### Statistical analysis

All data from western blot and flow cytometry analysis were representative of at least three independent experiments. Data were analysed with GraphPad Prism 6 Software to generate curves and bar charts. Statistical analyses were performed using Student *t* test (two tailed) or ANOVA (analysis of variance). The appropriate tests for multiple comparisons in ANOVA were specifically described in the corresponding Figure legends. *p < 0.05, **p < 0.01, and ***p < 0.001 were all considered statistically significant, while *p* > 0.05 was considered not significant (n.s).

## Electronic supplementary material

Figure S1

Figure S2

Figure S3

Figure S4

Figure S5

Figure S6

Figure S7

Figure S8

Figure S9

Figure S10

Figure S11

Figure S12

Figure S13

Figure S14

Figure S15

Figure S16

Supplements figure legends (clean)

Supplementary table 1

Supplementary table 2

Supplementary table 3
