# Supplementary material for: The proton pump inhibitor pantoprazole disrupts protein degradation systems and sensitizes cancer cells to death under various stresses
Source: Cell Death Dis. 2018 May 22;9(6):604. doi: 10.1038/s41419-018-0642-6 (PMC5964200; doi:10.1038/s41419-018-0642-6)
Supplement: Supplementary file 17 — Supplements figure legends (clean) [file 41419_2018_642_MOESM17_ESM.docx]

**Figure S1. PPI exhibited a dose and time dependent antitumor activity and promoted autophagy in different cancer cell lines.** (a) The morphological change of AGS and HeLa cells treated with PPI for 48 h in pH 7.4 condition. (b) CCK8 assay for determining cell viability after PPI treatment for 24 h and 48 h in pH 7.4 condition. (c) CCK8 assay for determining cell viability after PPI treatment for 48 h in pH 6.5 and pH 7.4 conditions. (d) Viable cell numbers counted by Trypan blue staining after PPI treatment for different times. (e) HGC27, MKN45, Panc-1, HCT116, Hucct1, A549 and U2OS were treated with indicated concentrations of PPI (60-140 μg/ml) for 48 h in pH 7.4 condition. Levels of related proteins were analyzed by western blot.

**Figure S2. PPI promoted autophagosome formation in neutral pH condition via Atg5 and Atg7**

(a) Autophagy-related proteins levels in cells after treated with PPI (60-140 μg/ml) for 48 h, were detected by western blot analysis. (b) The mRNA expression levels of autophagy-related genes after PPI treatment for 48 h were measured by qRT-PCR. Data were presented as mean ± SD (** p < 0.01, for each gene using one-way ANOVA Dunnett’s multiple comparison test). (c) AGS cells were transfected with Atg5, Atg7 and Beclin 1 siRNA separately for 48 h, or pretreated with the PI3K inhibitor (WM, 500 nM) for 2 h, and then incubated with 100 and 120 μg/ml PPI for another 48 h in pH 7.4 conditions. The level of indicated proteins were analyzed by western blot. (d) AGS cells transfected with GFP-LC3B plasmid were treated as described in (f), and then were observed under immunofluorescence microscopy. Scale bar: 20 μm. (e) GFP-LC3 dots assay. The average numbers of GFP-LC3 dots per cell in (g) were determined. Data were presented as mean ± SD from 3 independent experiments (n.s, not significant; ** p < 0.01, identified by two-way ANOVA with Tukey's multiple comparisons test). (f) HeLa and HGC27 cells were transfected with Atg5 and Atg7 siRNA separately for 48 h, and then incubated with indicated concentrations of PPI for another 48 h. The level of indicated proteins were analyzed by western blot. (g) HeLa and HGC27 cells were transfected with Beclin 1 siRNA for 48 h, or pretreated with the PI3K inhibitor (WM, 500 nM) for 2 h, and then incubated with indicated concentrations of PPI for another 48 h. The level of indicated proteins were analyzed by western blot.

**Figure S3. PPI also promoted autophagosome formation in acidic pH condition via Atg5 and Atg7**

(a) AGS cells treated with 100 μg/ml PPI for 48 h in pH 6.5 condition, were imaged by transmission electron microscopy. Representative micrographs showed increased abundance of lysosomes in cells after PPI treatment compared with control cells. Scale bar: 500 nm. (b) AGS and HeLa cells transfected with GFP-LC3B plasmid were treated with 100 μg/ml PPI for 48 h in pH 6.5 condition. Scale bar: 20 μm. (c) The number of autophagic vacuoles and GFP-LC3B dots in each cell were quantified. Data were presented as mean ± SD from 3 independent experiments (** p < 0.01, identified by Student's t-test). (d and e) Different cancer cells were treated for 48 h with indicated concentrations of PPI in pH 6.5 condition. Levels of LC3B-II and SQSTM1 protein were analyzed by western blot. β-actin served as an internal control. (f) The mRNA expression levels of autophagy-related genes after PPI treatment for 48 h in pH 6.5 condition were measured by qRT-PCR. Data were presented as mean ± SD (** p < 0.01, for each gene using one-way ANOVA Dunnett’s multiple comparison test). (g) AGS cells were transfected with Atg5, Atg7 and Beclin 1 siRNA separately for 48 h, or pretreated with the PI3K inhibitor (WM, 500 nM) for 2 h, and then incubated with 80 and 100 μg/ml PPI for another 48 h in pH 6.5 condition. The level of indicated proteins were analyzed by western blot.

**Figure S4.** **PPI inhibit the fusion of autophagosomes and lysosomes in acidic pH.** (a) AGS cells were either untreated or treated with PPI (40-120 μg/ml) for 24 h in pH 6.5 condition in the absence or presence of classical autophagic flux inhibitor baf-A1 (100 nM). The indicated protein levels were analyzed by western blot. (b) AGS cells were transiently infected with GFP-mRFP-LC3B adenoviral particles for 48 h and subsequently treated with PPI (100 μg/ml), baf A1 (100 nM), Torin 1 (500 nM) for 48h in pH 6.5 condition. The change of both green and red fluorescence was observed using a confocal microscope. Scale bar: 20 μm. Lower panel, the numbers of acidified autophagosomes (GFP^-^RFP^+^) versus neutral autophagosomes (GFP^+^RFP^+^) per cell in each condition were quantified. Data were presented as mean ± SD from 3 independent experiments (n.s, not significant; ** p < 0.01, identified by two-way ANOVA with Dunnett’s multiple comparison test). (c) Different cancer cells were either untreated or treated with PPI (60-120 μg/ml) for 48 h in pH 6.5 condition, and then stained with LysoTracker Red DND-99 dye (50 nM for 15 min) for FACS analysis. Representative results of 3 independent experiments were shown. (d and e) qRT-PCR analysis of various V-ATPase subunits in AGS (d) and HGC27 (e) cells treated with PPI for 48 h in pH 6.5 condition. Data were presented as mean ± SD.

**Figure S5. PPI induced lysosomal biogenesis and related lysosome genes in various cancer cell lines.** (a and b) The mRNA expression levels of various V-ATPase subunits in AGS cell line and TCGA STAD cohort (n=415). ATP6V0C served as a positive control. Data were presented as mean ± SD. (c and d) qRT-PCR analysis of various V-ATPase subunits (c) and lysosomal genes (d) in HGC27 cells treated with 60 and 100 μg/ml PPI for 48 h in pH 7.4 condition. Data were presented as mean ± SD. (e) The key lysosomal enzymes cathepsin D (CTSD) and cathepsin B (CTSB) were detected by western blot. (f) A549 cells were either untreated or treated with indicated concentrations of PPI (60-140 μg/ml) for 24 h in pH 7.4 condition in the absence or presence of rapamycin (1 μM) or baf-A1 (100 nM), then stained with LTR for FACS analysis. Representative results of 3 independent experiments were shown. (g) qRT-PCR analysis of various V-ATPase subunits in A549 cells treated with 60, 80 and 120 μg/ml PPI for 48 h in pH 7.4 condition. Data were presented as mean ± SD. (h) MKN45 cells were either untreated or treated with PPI (60-140 μg/ml) for 24 h in pH 7.4 condition in the absence or presence of baf-A1 (100 nM). The indicated proteins level were analyzed by western blot. (i) qRT-PCR analysis of various V-ATPase subunits in MKN45 cells treated with 60, 100 and 120 μg/ml PPI for 48 h in pH 7.4 condition. Data were presented as mean ± SD.

**Figure S6. PPI induced Nrf2 pathway activation following oxidative stress.** (a) Intracellular ROS generation induced by PPI in both pH 7.4 and pH 6.5 conditions was detected in HGC27 and HeLa cell lines. Data presented were representative of 3 independent experiments (** p < 0.01, identified by one-way ANOVA with Dunnett's multiple comparisons test). (b) PPI-induced cytotoxicity was dependent on ROS. After pretreatment with GSH (4 mM) or NAC (5 mM) for 4 h, AGS and HGC27 cells were incubated with 100 μg/ml PPI for 48 h in pH 7.4 condition. The cell viability was determined by CCK-8 (** P < 0.01, indentified by two-way ANOVA with Tukey's multiple comparisons test). (c) AGS cells were treated as in Fig 3c & 3d, the apoptosis marker cleaved PARP and the stress marker p-p38 were detected by western blot analysis. (d and e) HGC27 cells were exposed to various concentrations of PPI for 48 h in both pH 7.4 and pH 6.5 conditions. The levels of Nrf2 and its targets such as GCLC, GCLM, NQO1 and HO-1 were assessed by western blot analysis (d) and qRT-PCR analysis (e). Data were presented as mean ± SD (** p < 0.01, for each gene using one-way ANOVA Dunnett’s multiple comparison test). (f) Scatterplots showing the positive correlation between NFE2L2 and SQSTM1 mRNA expression in TCGA, GSE63089 and GSE27342. Pearson’s coefficient tests were performed to assess statistical significance. The TCGA result was were generated by GEPIA (<http://gepia.cancer-pku.cn/detail.php?clicktag=correlation>).

**Figure S7. PPI caused exogenously expressed HA-tagged SQSTM1 accumulation.** (a) AGS cells were transfected with HA-tagged SQSTM1 plasmid for 48 h followed by culture in PPI-containing medium for 48 h in both pH 7.4 and pH 6.5 conditions. Cells were examined by immunofluorescence microscopy using HA antibody to demonstrate exogenously expressed SQSTM1 (green).

**Figure S8. PPI induced SQSTM1 colocalized with Ubiquitin.** (a) AGS cells were transfected with HA-tagged Ub plasmid for 48 h followed by culture in PPI-containing medium for 48 h in both pH 7.4 and pH 6.5 conditions. Cells were examined by immunofluorescence microscopy using HA antibody to demonstrate Ub (green) or p62 antibody (Red).

**Figure S9. PPI reduced proteasome and immunoproteasome subunits expression.** (a) Cancer cells were treated with indicated concentrations of PPI for 48 h in pH 6.5 and pH 7.4 conditions, followed by measuring proteasome function via western blot analysis using specific antibody to ubiquitin. (b) The proteasome substrate TP53 was accumulated after PPI exposure for 48 h in pH 7.4 condition, and further increased when the MDM2 inhibitor nutlin-3a simultaneously added. (c) The mRNA expression level of the 20S proteasome subunits were measured after PPI treatment for 48 h in pH 6.5 condition. Data were presented as mean ± SD (* P < 0.05, ** P < 0.01, for each gene using one-way ANOVA with Dunnett’s multiple comparisons test).

**Figure S10. PPI may decrease proteasome and immunoproteasome subunits expression via inhibiting STAT3 pathway**

(a) The phosphorylated STAT3 (Y705) and its targets such as c-myc and cyclin D1 were measured after PPI treatment in both pH 7.4 and pH 6.5 conditions, respectively. (b) The mRNA expression levels of immunoproteasome subunits, including PSMB8 and PSMB9 with potential STAT3 binding sites in the promoter, were assayed by qRT-PCR. Data were presented as mean ± SD (** p < 0.01, for each gene using one-way ANOVA Dunnett’s multiple comparison tests). (c) The positive correlation between STAT3 and PSMB8/9 mRNA expression was confirmed in GSE63089 and GSE27342. Pearson’s coefficient tests were performed to assess statistical significance.

**Figure S11. PPI-induced ER stress was accompanied with perturbations in Ca^2+^ homeostasis.** (a) Western blot analysis of ER stress-related proteins after PPI treatment for 48 h in HGC27 and HeLa cells in pH 7.4 conditions. (b) qRT-PCR analysis of UPR target gene expression was performed after 48 h of PPI treatment in pH 7.4 conditions. Data were presented as mean ± SD (**P < 0.01, for each gene using one-way ANOVA with Dunnett's multiple comparisons test). (c) ER-Tracker Red (500 nM) staining of PPI treated cells in pH 6.5 condition was performed. The quantification of ER Tracker fluorescence was accomplished by FACS. Data presented were representative of 3 independent experiments (** P < 0.01, for each cell line using one-way ANOVA with Dunnett’s multiple comparisons test). (d) After treated with PPI (40-120 μg/ml) in pH 6.5 condition, cells were then incubated with 5 μM Fluo-4/AM and detected by flow cytometry. Data presented were representative of 3 independent experiments (** P < 0.01, identified by one-way ANOVA with Dunnett’s multiple comparisons test). (e) The mRNA expression levels of three types of ITPR in AGS cell line and TCGA STAD cohort (n=415). Data were presented as mean ± SD. (f) The mRNA change patterns of two key ER Ca^2+^ channels, SERCA and ITPR3, were determined by qRT-PCR analysis. Data were presented as mean ± SD (** P < 0.01, for each gene using one-way ANOVA with Dunnett's multiple comparisons test).

**Figure S12. PPI-induced ER stress was also dependent on protein synthesis level in low pH condition.** (a) AGS cells were pretreated with PPI (80 μg/ml) for 24 h, followed by combination with or without 25 nM Bortezomib for another 24 h in pH 6.5 condition. The cell viability was determined by CCK8 assay (left) (** P < 0.01, identified by two-way ANOVA with Sidak's multiple comparisons test). qRT-PCR analysis of UPR genes was performed (right) (** P < 0.01, for each gene using one-way ANOVA with Tukey's multiple comparisons test). (b) AGS cells were pretreated with CHX (50 ng/ml) for 2 h, and then incubated with PPI (80 μg/ml or 100 μg/ml) for 48 h in pH 6.5 condition. The cell viability was determined by CCK8 assay (left) (** P < 0.01, identified by two-way ANOVA with Sidak's multiple comparisons test). qRT-PCR analysis of UPR genes was performed (right) (** P < 0.01, for each gene using one-way ANOVA with Tukey's multiple comparisons test). (c) AGS cells were pretreated with torin 1 (250 nM) for 2 h, and then incubated with PPI (80 μg/ml or 100 μg/ml) for 48 h in pH 6.5 condition. The cell viability was determined by CCK8 assay (left) (** P < 0.01, identified by two-way ANOVA with Sidak's multiple comparisons test). qRT-PCR analysis of UPR genes was performed (right) (** P < 0.01, for each gene using one-way ANOVA with Tukey's multiple comparisons test).

**Figure S13. Proteasome inhibition by PPI was responsible for autophagy induction.** (a and b) AGS cells were treated as described in Fig. 6a, and the expression of LC3B-II was measured by western blot (a) and qRT-PCR (b). Data were presented as mean ± SD (** P < 0.01, identified by one-way ANOVA with Tukey's multiple comparisons test). (c and d) AGS cells were treated as described in Fig. 6g, and the expression of LC3B-II was measured by western blot (c) and qRT-PCR (d). Data were presented as mean ± SD (** P < 0.01, identified by one-way ANOVA with Tukey's multiple comparisons test). (e and f) AGS and HGC27 cells were pretreated with torin 1 (500 nM) or rapamycin (1 μM) for 2 h, and then incubated with indicated concentrations of PPI for 48 h in pH 7.4 condition. The expression of LC3B-II was measured by western blot (e) and qRT-PCR (f). Data were presented as mean ± SD (** P < 0.01, identified by one-way ANOVA with Tukey's multiple comparisons test).

**Figure S14. Autophagy acted as a protective response for proteasome inhibition by PPI.** (a) AGS and HGC27 cells were treated with 100 nM baf A1 and 50 μM HCQ for 24 h, and then the accumulation of poly-ubiquitinated proteins were analyzed by western blot. (b) AGS cells were transfected with Atg5 or Atg7 siRNA for 48 h, and then treated with 25 nM bortezomib for an additional 24 h. The indicated proteins were analyzed by western blot. (c) AGS, HGC27 and HeLa cells were transfected with Atg5 or Atg7 siRNA for 48 h in pH 7.4 condition, and then incubated with 100 and 120 μg/ml PPI for another 36 h. The apoptosis marker cleaved PARP was detected by western blot. (d) AGS and HGC27 cells were pretreated with 100 μg/ml PPI for 24 h, followed by combination with or without 50 μM HCQ for another 24 h in pH 7.4 condition. The indicated proteins were analyzed by western blot. (e) AGS cells were treated as described in (d). The accumulation of poly-ubiquitinated proteins were analyzed by western blot. (f and h) ER-Tracker Red (500 nM) staining of single PPI treated cells in pH 7.4 and pH 6.5 condition (f), and PPI combined with HCQ treated cells in pH 7.4 condition (h) was performed. The quantification of ER Tracker fluorescence was accomplished by FACS. Data presented were representative of 3 independent experiments (** P < 0.01, for each cell line using one-way ANOVA with Dunnett’s multiple comparisons test). (g and i) Q-PCR analysis for UPR markers in PPI treated cells in pH 7.4 and pH 6.5 condition (f), and PPI combined with HCQ treated cells in pH 7.4 condition (h) (** P < 0.01, for each gene using one-way ANOVA with Tukey's multiple comparisons test).

**Figure S15. PPI synergized the antitumor activity of Bcl-2 inhibitors.** (a) AGS cells were treated with PPI (60-140 μg/ml) for 24 h in both pH 6.5 and pH 7.4 condition, and then stained with MitoSox Red probe to determine the mitochondrial ROS by flow cytometry. Data were representative of 3 independent experiments (** p < 0.01, indentified by one-way ANOVA Dunnett’s multiple comparisons test). (b) AGS cells were treated as described in (a), and then incubated with 4 μM Rhod-2/AM for 30 min for flow cytometry. Data were representative of 3 independent experiments (** p < 0.01, indentified by one-way ANOVA Dunnett’s multiple comparisons test). (c) HGC27 cells were pretreated with various concentrations of PPI for 24 h, and then incubated with two different doses of ABT-263/ABT-737 for another 24 h in pH 7.4 condition. Data were presented as percentages of cell viability as determined by CCK8 assays (upper panel). Synergisms of cell viability inhibition by the combination therapy were analyzed by Combination Index Value (lower panel). (d) Cell viability of HGC27 cells after treatment with combination of 100 μg/ml PPI and 4 μM ABT-263/10 μM ABT-737 in pH 7.4 condition. The combination indexes (CI) were calculated as described in Methods. Data were presented as mean ± SD (*** p < 0.001, indentified by one-way ANOVA Dunnett’s multiple comparison test). (e and f) AGS and HGC27 cells were treated as described in Fig. 7c & Fig. S15c, and the percentage of PI positive cells were quantified by FACS. Right panel showed the quantification of the percentage of PI positive cells. Data were presented as mean ± SD (n.s, not significant; *** p < 0.001, indentified by one-way ANOVA Tukey's multiple comparisons test). (g) HGC27 cells were treated as described in (c), and the changes in mitochondrial membrane potential (Δψm) were analyzed by JC-1 assay. Ratio of green to red fluorescence was depicted (left panel). Right panel showed the quantification of the Δψm. Data were presented as mean ± SD (** p < 0.01, *** p < 0.001, indentified by one-way ANOVA Tukey's multiple comparisons test).

**Figure S16. PPI also synergized the antitumor activity of Bcl-2 inhibitors in low pH condition.** (a) AGS cells were pretreated with various concentrations of PPI for 24 h, and then incubated with two different doses of ABT-263/ABT-737 for another 24 h in pH 6.5 condition. Data were presented as percentages of cell viability as determined by CCK8 assays (upper panel). Synergisms of cell viability inhibition by the combination therapy were analyzed by Combination Index Value (lower panel). (b) Cell viability of AGS cells after treatment with combination of 80 μg/ml PPI and 2 μM ABT-263/2.5 μM ABT-737. The combination indexes (CI) were calculated as described in Methods. Data were presented as mean ± SD (*** p < 0.001, indentified by one-way ANOVA Dunnett’s multiple comparison test). (c) HGC27 cells were pretreated with various concentrations of PPI for 24 h, and then incubated with two different doses of ABT-263/ABT-737 for another 24 h in pH 6.5 condition. Data were presented as percentages of cell viability as determined by CCK8 assays (upper panel). Synergisms of cell viability inhibition by the combination therapy were analyzed by Combination Index Value (lower panel). (d) Cell viability of HGC27 cells after treatment with combination of 60 μg/ml PPI and 2 μM ABT-263/2.5 μM ABT-737 in pH 6.5 condition. The combination indexes (CI) were calculated as described in Methods. Data were presented as mean ± SD (*** p < 0.001, indentified by one-way ANOVA Dunnett’s multiple comparison test).
